# Glycosylation and behavioral symptoms in neurological disorders

**DOI:** 10.1038/s41398-023-02446-x

**Published:** 2023-05-08

**Authors:** Prajitha Pradeep, Hyeyeon Kang, Boyoung Lee

**Affiliations:** 1 Center for Cognition and Sociality, Institute for Basic Science, Daejeon, 34126 South Korea; 2 IBS School, University of Science and Technology (UST), Daejeon, 34113 South Korea; 3 Department of Biomedical Engineering, College of Information and Biotechnology, Ulsan National Institute of Science and Technology (UNIST), Ulsan, 44919 South Korea

**Keywords:** Neuroscience, Diseases

## Abstract

Glycosylation, the addition of glycans or carbohydrates to proteins, lipids, or other glycans, is a complex post-translational modification that plays a crucial role in cellular function. It is estimated that at least half of all mammalian proteins undergo glycosylation, underscoring its importance in the functioning of cells. This is reflected in the fact that a significant portion of the human genome, around 2%, is devoted to encoding enzymes involved in glycosylation. Changes in glycosylation have been linked to various neurological disorders, including Alzheimer’s disease, Parkinson’s disease, autism spectrum disorder, and schizophrenia. Despite its widespread occurrence, the role of glycosylation in the central nervous system remains largely unknown, particularly with regard to its impact on behavioral abnormalities in brain diseases. This review focuses on examining the role of three types of glycosylation: N-glycosylation, O-glycosylation, and O-GlcNAcylation, in the manifestation of behavioral and neurological symptoms in neurodevelopmental, neurodegenerative, and neuropsychiatric disorders.

## Introduction

**Fig. 1:**
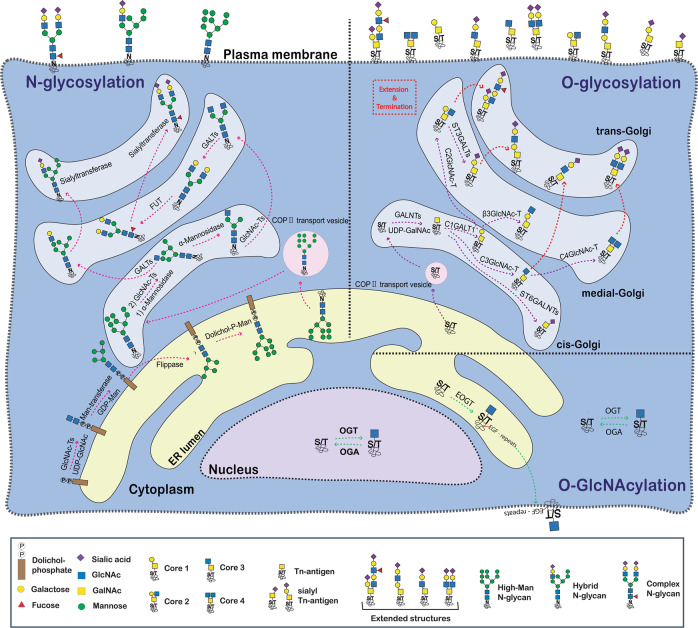
Graphic summary for the comprehensive overview of glycosylation. The summary covers the three main types of glycosylation and a simplified biosynthetic pathway for each type.

Glycosylation is the most abundant and complex but highly conserved essential modification in which a carbohydrate consisting of several sugar molecules, sometimes also referred to as a glycan, is attached to a protein, lipid, or glycan substrate [1]. There are two major types of protein glycosylation in eukaryotes: N-glycosylation (N-linked glycosylation) and O-glycosylation (O-linked glycosylation) (Fig. 1) [1, 2]. N-glycosylation is known to be initiated in the endoplasmic reticulum (ER) and further modifications are processed in the Golgi apparatus [3]. In contrast to N-glycosylation, the biosynthesis of O-glycosylation is mostly initiated in the Golgi apparatus through the stepwise addition of monosaccharide residues, although the initiation of some O-glycans occurs in ER [2]. Many glycans found on the outer surface of a cell, mostly assembled through the ER–Golgi pathway through N- or O-glycosylation, manage cell‒cell, cell‒matrix, or cell‒molecule interactions that regulate diverse cell functions [1, 2] Within O-glycosylation, O-GlcNAcylation is unique in terms of the location of the target substrates such as the nucleus, mitochondria and cytosol (Fig. 1) [4, 5]. O-GlcNAcylation is rather simple but highly dynamic [4, 6] by competing reciprocally with phosphorylation and influences the regulation of nutritional state and cellular stress and has important effects on many cellular processes, such as cellular signaling, protein stability, transcription, and others [1, 2]. Therefore, it can be said that glycans are involved in almost all physiological processes. Thus, abnormal protein glycosylation can affect diverse physiological functions of the body, leading to various clinical symptoms.

Glycans are formed by a set of competing glycosyl transferases. Nearly 2% of the human genome encodes glycosyltransferases, glycosidases, or other glycan-modifying enzymes [7], and approximately half of all mammalian proteins are glycosylated, suggesting the importance of glycosylation in cellular function [8, 9]. Congenital disorders of glycosylation (CDGs) are a group of diseases caused by the lack of glycosylation enzymes and are known as a type of genetic abnormality. More than 150 subtypes of CDGs have been reported [10, 11]. The severity of CDGs may depend on the stages it affects; some affect a single glycosylation step, while others may affect the entire pathway [12]. Therefore, defects in glycosylation-related enzymes may lead to the development of rare but severe diseases. Glycosylation is particularly important in the brain as the majority of CDGs show neurological symptoms including psychomotor retardation, cognitive disorders, epileptic seizures, hypotonia, ataxia, polyneuropathy, and stroke-like events [13–15]. Diagnosing CDG is often difficult due to the wide range of clinical features affecting multiple organ systems and the phenotypic heterogeneity between CDG types [11, 16]. Therefore, studies of the function of each enzyme involved in glycan biosynthesis and its connection to clinical features can aid in developing phenotype-specific biomarkers or treatment targets.

The diversity of glycomes and glycoproteomes is greatly increased by the various combinations of monosaccharides [17]. Recent advances in high-throughput glycomic technology have enabled systematic analysis of glycomes in preclinical and clinical studies, revealing abnormal glycan structures as hallmarks of cancer [18]. Thus, altered glycomes are considered potential biomarkers or therapeutic targets for many disorders[19, 20]. Importantly, understanding glycan function is of great importance in the CNS, where differential glycan expression has recently been found in various stages of CNS diseases such as schizophrenia and Alzheimer’s disease (AD) [21–28]. However, the role of glycan alterations in glycoproteins and the function of glycoproteins in the CNS remain largely unknown, especially in relation to behavior abnormalities in CNS diseases. In this review, we discuss different types of glycosylation, specifically N-glycosylation, O-glycosylation, and O-GlcNAcylation, in relation to behavioral and neurological symptoms of neurodevelopmental, neurodegenerative, and neuropsychiatric disorders.

## N-Glycosylation

N-glycosylation involves the attachment of N-glycans (N-acetylglucosamine/GlcNAc) to glycoproteins via the nitrogen atom of the Asn residue at Asn-X-Ser/Thr sites. It can be grouped into three classes, as oligomannose, complex, and hybrid N-glycans, based on the extensions. Oligomannose (or high-mannose) types are composed of a core extended solely by Man residues, complex N-glycans feature a core extended by GlcNAc residues, and hybrid N-glycans have a core extended by both Man and GlcNAc residues [29]. This process is important for the proper functioning and localization of proteins, and alterations in N-glycosylation have been linked to several brain disorders such as Alzheimer’s disease, Parkinson’s disease, and others [30]. In this section, we summarize the implications of abnormal N-glycosylation in brain disorders and the behavioral symptoms it associates with neurological disorders.

### Congenital disorders of glycosylation (CDG) of N-glycosylation

CDGs stands for Congenital Disorders of Glycosylation, a group of inherited metabolic disorders that result from defects in the synthesis and/or processing of complex sugars. These disorders can affect multiple systems in the body, including the brain, and some forms are associated with neurological symptoms and behavioral abnormalities. The most common type of N-glycosylation-related CDG, classified as Type I, is phosphomannomutase 2 or PMM2-CDG, which results from a mutation of the *PMM2* gene that converts mannose-6-phosphate (M6P) to mannose-1-phosphate (M1P) [12]. The effects of the mutation range from embryonic lethal cases to mild cognitive impairments [1, 12]. Patients, especially infants with PMM2-CDG, have an underdeveloped cerebellum (cerebellar atrophy) that coordinates movement [31, 32]. In a human study, disrupted N-glycosylation of Ca_V_2.1 channels was related to cerebellar ataxia and stroke-like episodes in PMM2-CDG patients [33].

Next to PMM2-CDG, the most common type of CDG is Man-6-P isomerase or MPI-CDG [1, 12]. Patients exhibit delayed growth and suffer from liver and gastrointestinal diseases, but they have normal intellectual development [34]. SLC35A2-CDG is another well-studied type of CDG in which changes in N-glycosylation lead to neurological symptoms, especially epilepsy, developmental delay, and intellectual disability [35–46]. *SLC35A2* codes the X-linked UDP-galactose transporter that commutes UDP galactose from the cytosol to the Golgi. Human patients with SLC35A2-CDG showed reduced galactose and truncated glycans [34, 35]. DPAGT1-CDG, a very rare type of CDG, is caused by mutations in the *DPAGT1* gene leading to disrupted neurotransmission, long-term muscle weakness, and multisystem breakdown [34]. *DPAGT1* encodes UDP-*N*-acetylglucosamine-dolichol-phosphate-*N*-acetylglucosamine-phosphotransferase. Mass spectrometry analysis of serum transferrin from human patients with DPAG1-CDG revealed abnormal N-glycosylation [47, 48]. In addition, *DPAGT1* homozygous knockout mouse models were found to be embryonic lethal. Other N-glycosylation-related CDGs include ALG1-CDG, which results in intellectual disability, developmental delay, and epilepsy [49]; NGLY1-CDG, which causes intellectual disability and alacrimia [34]; and SRD5A3-CDG, which causes severe developmental disability, skin problems, cerebellar ataxia and ocular anomalies [34, 50]. The CDGs listed here are CDGs that display phenotypic features associated with neurological disorders. The discovery of CDGs associated with behavioral abnormalities in humans highlights the crucial role of N-glycosylation in brain function.

### Neurodevelopmental disorders

Autism spectrum disorder (ASD) is a group of neurodevelopmental disorders with problems in social interaction, delayed speech, nonverbal communication, repetitive behavior, and restrictive interests [51]. Not much is known about the role of N-glycosylation in ASD. A recent study with an ASD patient, focused on the aberrant N-glycosylation of X chromosome-linked neuroligin 4 or NLGN4, a member of the family of *NLGN* genes associated with ASD. The study showed a mutation at the R101Q residue in NLGN4 that impaired normal N-glycosylation at its adjacent N102 residue, which affected NLGN4 surface trafficking and led to synaptic dysfunction in NSC-derived neurons [52].

Childhood disintegrative disorder (CDD) is considered to be a rare form of ASD. A study focused on N-glycans in patients with late-onset Tay-Sachs disease (LOTSD), a type of CDD. Patients with LOTSD are generally infants younger than a year and show hexosaminidase A enzyme deficiency, which causes abnormal accumulation of GM2 ganglioside in neurons, accelerating neuron deficiency [53]. Studies identified a lack of sialylation in brain N-linked glycoprotein, but no changes were found in plasma N-glycans in patients. The abnormal accumulation of GM2 ganglioside was found to be linked with an impaired turnover of membrane glycolipids in the endocytotic-lysosomal pathway, further affecting glycosyltransferase activities [53]. Patients suffer from loss of sensory and motor function, epilepsy, cognitive impairment, or early mortality [53].

In another study, human plasma N-glycome analysis was performed in patients with attention-deficit hyperactivity disorder (ADHD). The symptoms of ADHD include restlessness, hyperactivity, disrupted social interactive skills, poor time management, and inability to be attentive. Plasma N-glycan analysis revealed a significant increase in biantennary glycans with antennary fucose (A2FG2) and a significant decrease in tri- and tetra-antennary glycans in ADHD patients [54].

The evidence linking N-glycosylation abnormalities and neurodevelopmental disorders is increasing, but more mechanistic studies are needed to identify targets and effects of abnormal N-glycosylation on their targets to fully understand the roles of N-glycosylation in neurodevelopmental disorders, such as ASD and ADHD.

### Neurodegenerative disorders

Alzheimer’s disease (AD) is one of the most common neurodegenerative disorders that affect memory, thinking, and cognitive abilities. It begins asymptomatically but slowly progresses to mild cognitive impairment followed by different stages of dementia that worsen over time, interfering with the patient’s daily life [55]. Aberrant N-glycosylation is considered to be a potential biomarker for Alzheimer’s disease progression [56]. Increases in bisected and branched N-glycans have been identified in the serum and cerebrospinal fluid (CSF) of Alzheimer’s disease patients [57]. In addition, many Aβ-related molecules are known to be N-glycosylated. For example, disruption in the N-glycosylation of BACE1, an Aβ-producing β-secretase, affects the folding and maturation of the protein, disrupting its downstream pathways, which can be linked to AD development [58, 59]. Others include amyloid precursor protein (APP), Nicastrin (γ-secretase subunit) [60, 61], ADAM10 (a protease), Neprilysin (Aβ-degrading enzyme), and TREM2 (triggering receptor expressed on myeloid cells 2). However, the underlying mechanisms of abnormal glycosylation on these proteins are still unknown [59]. Tau protein aggregation is a well-known hallmark of AD. The cytosolic protein tau is well known to be glycated and O-GlcNAcylated [62] but not N-glycosylated [59]. Interestingly, in AD, tau was found to be N-glycosylated at the N410 site on the tau 2N4R isoform [63], which precedes abnormal phosphorylation on tau. Thus, this abnormal N-glycosylation on tau may have the potential as an earlier biomarker of AD, as well as a marker of AD progression. Furthermore, in an N-glycoproteomics analysis specific to AD pathophysiology, 137 differentially N-glycosylated (hyperglycosylated and hypoglycosylated) proteins and 178 aberrant N-glycosylation sites were reported [64]. Different levels of glycan expression were observed in the cortex and hippocampus between normal and AD-affected brains, and different glycan profiles in CSF were also observed in AD patients compared to healthy controls [65].

Parkinson’s disease (PD) is another well-studied neurodegenerative disorder. It is commonly characterized by the death of dopaminergic neurons and the existence of Lewy bodies as α-synuclein aggregates at the substantia nigra pars compacta. Patients show disruption of normal motor and nonmotor functions, such as rigidity, bradykinesia, sleep disruption, depression, and cognitive decline. Similar to AD, aberrant protein glycosylation is found in PD progression, but little is known about the role of N-glycosylation in PD. Some of the PD-specific proteins that are proposed to have abnormal glycosylation include the receptor for advanced glycation end products (RAGE), which has two *N*-glycosylation sites and are considered to influence RAGE signaling by affecting its binding to other molecules, and TREM2, which showed changes in *N*-glycans, including sialylated and/or fucosylated complex-type glycans [66]. Dopamine transporter (DAT) was found to be N-glycosylated which is involved in the cell surface expression of DAT and dopamine transportation. Aberrant glycosylation of DAT disrupts its activity, leading to dopamine accumulation in the extracellular space of SH-SY5Y cells [67]. In a recent N-glycoproteomics-based study, researchers identified an increase in the number of glycans containing core fucose, sialic acid, and bisecting N-acetylglucosamine in the sera of human patients, both at the overall glycan level and at specific glycosites of proteins related to Parkinson’s disease. These proteins include Ceruloplasmin, Haptoglobin, Kininogen-1, Complement Factor H, and Clusterin [68].

Multiple sclerosis (MS) is a neurodegenerative and autoimmune disorder in which the immune system attacks nerve fibers and myelin in the CNS, interfering with normal signal propagation. Symptoms vary among patients and can include numbness, tingling, memory, cognitive impairment, pain, fatigue, blindness (blurred, doubled, or complete loss of vision), balance or coordination issues, and paralysis. Defective N-glycosylation is considered to directly affect innate immune activity and neurodegeneration [56]. N-glycome analysis of plasma and IgG showed altered N-glycosylation in MS. Earlier research on mouse models showed that GlcNAc branching deficiency affects T-cell activity, autoimmune responses, and apoptosis in neurons [69]. Human studies have shown decreased branching and fucosylated N-glycans in MS [70]. Myelin oligodendrocyte glycoprotein (MOG), a primary target autoantigen in an animal model of MS, is also known to have one N-glycosylation site at N31, and mutation at this site has led to autoantibody recognition in MS patients [71].

N-glycosylation alterations have been identified in various neurodegenerative disorders, including AD, PD, and MS, suggesting the role of N-glycosylation in the development or progression of these conditions. However, although aberrant glycosylation has been identified in certain glycoproteins, it is still unclear how to regulate it with current techniques to rescue altered glycosylation and associated symptoms. Therefore, further studies are required to understand the relationship between altered N-glycosylation and the mechanisms underlying neurodegenerative diseases, including AD, PD, and MS.

### Neuropsychiatric disorders

Schizophrenia is a neuropsychiatric disorder affecting a person’s thoughts, feelings, and behavior [72]. Although the cause remains unclear, a combination of physical, genetic, and environmental factors can explain the condition. Symptoms can be positive (hallucinations and delusions), negative (lack of motivation and interest, isolation, and difficulty in expressing emotions), and cognitive (lack of attention and disrupted decision-making) [72–75]. Changes in N-glycosylation have been reported in the serum and CSF of schizophrenic patients [76]. Several variations in the normal expression of the N-glycans across different types of glutamate receptors, such as the AMPA receptor subunit (GluA2) and kainate receptor subunit (GluK2), in neurons and glutamate transporters, such as excitatory amino acid transporter 1 (EAAT1) and excitatory amino acid transporter 2 (EAAT2), predominantly expressed in astrocytes, were observed in schizophrenic patients [7, 77–79]. Abnormal N-glycosylation was also found across different GABA receptor subunits (GABA_A_ receptors (α1, β1, and β2)) in schizophrenia, indicating that disruptions of N-glycosylation in schizophrenia are not limited to glutamate and may affect the central process for neuronal communication in this disorder [7, 77, 79, 80]. Recently, it has been reported that the schizophrenia risk locus *SLC39A8* (also known as *ZIP8*) is involved in N-glycosylation. SLC39A8 is a transmembrane protein that cotransports bicarbonate and divalent cations, specifically transporting Mn in cells. Thus, homozygous loss-of-function mutations in *SLC39A8* result in low plasma manganese (Mn). Importantly, since manganese is involved in the activity of enzymes related to N-glycan biosynthesis, N-glycan profiles have been analyzed, and increased precursor N-glycans and decreased complex N-glycans have been identified in individuals with CDGs caused by homozygous *SLC39A8* mutations [81]. In summary, studies tackling the link between glycosylation and schizophrenia have allowed us to understand the importance of N-glycosylation in pathogenesis and to identify novel therapeutic targets and biomarkers for schizophrenia [79, 81].

Post-traumatic stress disorder (PTSD) is a neuropsychiatric disorder that occurs in people who experience or witness traumatic events. Symptoms include repeated memories and distressing visuals, changes in mood (irritability, anger outbursts, and fear), and abnormal cognition. In a very recent study, an alteration in plasma N-glycans was discovered in PTSD patients along with accelerated physiological aging determined with the GlycoAge Test index [82]. Research on rat PTSD models also showed alterations in N-glycans. The rats showed high-freezing behavior and decreased social interest. The authors suggested that the simplification of large and elaborate N-glycans, especially in the prefrontal cortex, is associated with the pathology of PTSD and can be considered candidates for trauma-related biomarkers [83].

Depression is a serious mood disorder that negatively affects feelings, thoughts, and actions. People with depression feel sad, hopeless, frustrated, and worthless along with experiencing decreased energy, difficulty sleeping or waking up and concentrating. Bipolar disorder is a mental disorder with changes in mood, energy, and normal activities. The patients may experience manic or depressive episodes. Manic episodes elevate the feelings, leading to hyperactivity and loss of appetite, while depressive episodes can lead to a depressed mood (feeling sad), increased appetite, trouble sleeping or concentrating. Few studies have focused on the role of N-glycosylation in depression and bipolar disorders. Recent research on female patients with major depressive disorder showed alterations in serum N-glycans [84]. This alteration was associated with the disease severity and the expression of inflammatory markers. Patients with acute MDD showed decreased galactosylated N-glycans and increased tri-antennary N-glycans in correlation with increased serum IL-6 and CRP levels [84]. A similar sex-specific effect of altered plasma N-glycans was found by another research group, suggesting its potential use as a biomarker [85]. In a very early study, patients suffering from bipolar affective disorder showed disruption in the *DIBD1* gene (alpha-1,2-mannosyltransferase), which catalyzes the transfer of mannose from Dol-P-Man to lipid-linked oligosaccharides, the first step of lipid-linked N-glycan assembly [86]. Although the research suggested that disruption in *DIBD1* may affect the subsequent posttranslational modifications of the proteins, they could not confirm its role in the cause of the disease [86].

Studies on N-glycosylation in neuropsychiatric diseases have not progressed as much as in other diseases. Nevertheless, recent findings show that abnormal N-glycosylation is present in several neuropsychiatric disorders such as schizophrenia, PTSD, and MDD, attracting attention as potential new biomarkers.

## O-glycosylation

O-glycosylation (or O-linked glycosylation) is a highly common posttranslational modification of proteins, with important clinical significance in physiology and diseases. O-glycosylation is the addition of O-glycans, especially N-acetylgalactosamine or GalNAc, to the Ser and Thr hydroxyl residues of glycoproteins [12, 87]. O-Glycans form six major core structures and later extend linearly or with branching [1]. Mucin-type O-glycosylation is the most common type of O-glycosylation and is initiated in the Golgi apparatus by a family of ~20 enzymes known as UDP-GalNAc:polypeptide N-acetylgalactosaminyltransferases (GalNAc-Ts or GALNTs). This process is also termed O-GalNAcylation [1, 87]. Discovered in the late 1800s, mucin-type O-glycosylation was found to modulate secreted and membrane-bound proteins throughout the body. It is involved in diverse functions, such as cell‒cell communication, cell adhesion, host‒pathogen interactions, and immune surveillance [12, 87]. Abnormal O-glycosylation is known to be associated with a variety of human diseases, such as tumor progression and dysregulation of the immune system or metabolism [12]. However, little is known about O-glycosylation in brain disorders. Their role is being studied widely in Alzheimer’s, autism spectrum disorder, and schizophrenia. Here, we mainly focused on abnormalities of aberrant mucin-type O-glycosylation in brain disorders.

### CDG of O-glycosylation

To date, ~30% of more than 100 human CDGs are present in the O-glycosylation pathway [88, 89]. CDGs of O-glycosylation affect cellular function in multiple organs and exhibit a variety of clinical manifestations, including neurological impairments [90–92]. A recent study has shown that the loss of function of *GALNT2* in patients and rodent models leads to comprehensive developmental delay, intellectual disability, and abnormal behaviors such as autistic-like phenotypes and social problems [93]. Although GALNT2 is ubiquitously expressed in most cells of the body, it is implied that GALNT2 is important for normal brain function [93]. There are other types of CDGs in O-glycosylation other than mucin-type O-glycosylation. Defects in protein O-mannosylation lead to α-dystroglycanopathies, which cover a wide spectrum of disease severities that include neurodevelopmental disorders and progressive muscular dystrophy. In particular, deficiencies in the O-Man-transferases 1 and 2 (*POMT1* and *POMT2*, respectively) are associated with Walker-Warburg syndrome, and affected individuals may present with severe cognitive deficits, hydrocephaly, and brain and retinal dysplasia [94]. In addition, a defect in β1-2 GlcNAc-transferase (*POMGNT1*) has also been related to muscle–eye–brain disease characterized by congenital muscular dystrophy (CMD), ocular abnormalities and brain malformation (type II lissencephaly) [89, 95, 96]. Another CDG of O-glycosylation is Peters-Plus syndrome caused by mutations in β1-3 Glc-transferase (*B3GLCT*), which transfers glucose to O-linked fucose on thrombospondin type-1 repeats [89, 97]. Patients with Peters-Plus syndrome have several morphological anomalies, including Peters ocular malformation, disproportionate short stature, brachial dystrophy, dysmorphic facial features, and developmental delay [97]. The CDGs mentioned above are also accompanied by mental retardation and intellectual disability, highlighting the crucial role of O-glycosylation in maintaining normal brain function and behavior.

### Neurodegenerative diseases

The dysregulation of glycans has been involved in a number of neurodegenerative diseases, such as AD, PD, and Huntington’s disease or HD [95, 98]. In a recent study, glycoproteomic techniques provided perspectives on the significant role of glycosylation in neurodegenerative diseases [99]. Among the various types of O-glycosylation, mucin-type O-glycosylation has been extensively studied in neurodegenerative diseases. For example, several GLANTs have been shown to transfer GalNAc to APP (amyloid-β precursor protein), which may be involved in APP cleavage and Aβ production in AD [100]. Furthermore, the mRNA expression levels of *GALNTs* (*GALNT4, 6* and *10*) were significantly increased in the brains of AD patients, supporting an association of mucin-type O-glycosylation in AD development [100]. However, another in vitro study showed that the excessive O-glycosylation of APP by GALNT6 significantly reduced Aβ production [101]. While the role of O-glycosylation on APP in AD pathogenesis is still unclear, it is evident that dysregulated O-glycosylation is associated with AD. Further investigation into the glycosylation sites by different GALNTs on APP and the role of O-glycosylation in Aβ production is necessary.

HD is a rare and inherited neurodegenerative disease. It is characterized by progressive neurodegeneration, motor impairment, and decreased cognitive function due to mutation of the huntingtin (*HTT*) gene coding its protein HTT. Previously, extensive glycome profiling in HD transgenic mice showed that the most abundant mucin type of O-glycans in brain tissue was the core 3 type (GlcNAcβ1-3GalNAcαSer/Thr), which was differentially expressed in a sex-specific manner [102]. It is found to be increased in males and decreased in females [102]. Therefore, mucin-type O-glycosylation could be a promising biomarker for a gender-specific manner in HD pathophysiology.

In demyelinating diseases such as MS, the absence of branched O-mannosylation in astrocytes enhanced remyelination in the cuprizone-induced demyelinating mouse model, indicating that the modulation of O-mannosyl glycans is probably a vital player in the regulation of remyelination in MS [103].

### Neurodevelopmental and neuropsychiatric diseases

Abnormal O-glycosylation is also known to contribute to neurodevelopmental and neuropsychiatric disorders such as ASDs, schizophrenia, and depression [79, 104]. According to genome-wide association studies (GWAS), a subset of genes encoding O-glycosylation enzymes have been found to be highly associated with ASD and schizophrenia [51, 105]. For example, *GALNT9*, which is mainly expressed in the brain, was reported to be associated with ASD [51, 105, 106]. In clinical studies, microdeletions of chromosome 2q have also been reported in ASD individuals with developmental delay, language delay, communication disorders, and social anxiety [107, 108]. *GALNT13* is one of the genes located in a microdeletion on chromosome 2q. Indeed, GALNT13 is specifically expressed in the brain and has an important role in neural development, suggesting a possible association with ASD [109].

GALNT10, which is widely expressed in the body, including the brain, was found to be strongly associated with schizophrenia [105]. In addition, a postmortem study determined that the protein level of GALNT16 was significantly decreased in the superior temporal gyrus tissues in schizophrenia [110].

With regard to major depressive disorder (MDD), also called clinical depression, which is characterized by various emotional and physical problems, microarray-based profiles in the postmortem brain tissue of MDD patients identified upregulation of the GALNT family [111]. In particular, microarray analysis of MDD showed that *GALNT2* and *GALNT12* were increased in the striatum but decreased in the hippocampus, implying that they might have different functions in different brain regions in MDD progression [112]. However, most of this evidence comes from association studies, and there is no direct evidence that O-GalNAcylation is involved in regulating multiple social behaviors, including ASD, schizophrenia, and depression. More research is needed to address this question.

Compared to N-glycosylation, research on O-glycosylation in brain diseases is scarce. Most studies have centered on glycosyltransferases expression, such as GALNTs, instead of O-glycan structures or their downstream targets. Hence, high-throughput glycomics and glycoproteomics are required, using post-mortem brain samples from patients or preclinical animal models, to gain insight into the specific mechanisms behind abnormal O-glycosylation in disease pathology and to identify new biomarkers or potential treatment targets.

## O-GlcNAcylation

Another major type of O-glycosylation is a modification by O-GlcNAc or O-linked β-*N*-acetylglucosamine to serine or threonine hydroxyl residues on nuclear, mitochondrial, and cytoplasmic proteins and is termed O-GlcNAcylation. Not found until 1983, O-GlcNAc was found to be localized in the nuclear pore and within the cytoplasm [113]. Unlike other types of glycosylation, the O-GlcNAc modification is not elongated or modified. It is a very dynamic process involving two glycosyltransferases, O-linked GlcNAc transferase (OGT) and O-GlcNAcase (OGA). This dynamic and rapidly reversible cycle involves the addition of O-GlcNAc to the proteins and the removal of the same [1]. It was also found that O-GlcNAc modification competes with protein phosphorylation at the same site. Both OGT and OGA have only a single catalytic subunit, but they exist as different holoenzymes within the cell. Interestingly, they are also modified by phosphorylation, nitrosylation, ubiquitination, and O-GlcNAcylation itself [113]. The overall levels of these enzymes are controlled by the cellular O-GlcNAc levels. At higher O-GlcNAc levels, OGA increases while OGT declines, and vice versa; thus, this pathway is referred to as the nutrient-sensitive pathway [113].

The notion of O-GlcNac modification specifically to the intracellular proteins was rewritten when in 2008, Matsuura et al. proposed that O-GlcNAc modification was also found in the extracellular environment on the epidermal growth factor (EGF) repeats [114] and Sakaidani et al. [115] reported the existence of a novel transferase responsible for extracellular O-GlcNAcylation termed as EOGT or EGF domain O-GlcNAc transferase in Drosophila models [115]. It was reported that both OGT and EOGT controlled their actions independently without affecting each other [115]. EOGT transfers GlcNAc to serine or threonine residues in secreted and membrane proteins that contain EGF-like repeats such as the Notch receptor, thus regulating normal development [115, 116]. Mutations were shown to result in abnormal cell–matrix or cell–cell interactions [115].

O-GlcNAcylation plays a large role in maintaining normal cellular functions such as transcriptional regulation, epigenetics, protein folding, localization and degradation, mitochondrial function, and metabolic and circadian regulation [113]. OGT and OGA are ubiquitously expressed in all tissues and have a high expression in the brain. More than 1000 O-GlcNAcylation protein targets have been discovered, and O-GlcNAcylated proteins are particularly abundant in the brain [117]. More importantly, more than 19% of all synaptosomal proteins regulating synaptic function were found to be O-GlcNAcylated [118] and abnormal O-GlcNAcylation of these proteins can lead to several neurological disorders [117, 118]. This section summarizes the pathophysiological role of abnormal O-GlcNAcylation in brain disorders.

### CDG of O-GlcNAcylation

Adams–Oliver syndrome (AOS) is a rare congenital disorder with congenital scalp defects (aplasia cutis congenita (ACC) and terminal transverse limb defects. Patients also have Intellectual disabilities, seizures, congenital heart defects, brain defects, and eye defects. Recent research reported that autosomal recessive (one of the modes of inheritance) Adams-Oliver syndrome has mutations in DOCK6 and EOGT genes [119]. Another study of mutations in Drosophila ogt demonstrated that a novel congenital glycosylation disorder (OGT-CDG) is associated with Intellectual disability [120]. In this study, an imbalance of O-GlcNAc cycling and catalytic deficiency of Ogt cause abnormal locomotor functions and habituation learning, leading to cognitive deficits and Oga deletion can rescue these deficits, as evidenced by improvements in habituation learning [120].

### Neurodevelopmental disorders

Rett syndrome (RTT) is a neurodevelopmental disorder that is classified as an autism spectrum disorder [121]. Clinically, RTT is characterized by psychomotor regression with loss of volitional hand use and spoken language, the development of repetitive hand stereotypies, and gait impairment [121, 122]. Mutations in the gene MECP2 is considered to be one of the causes of RTT. O-GlcNAcylation defects at the threonine 203 site in the mutations of the X-linked methyl-CpG-binding protein 2 (*MECP2*) gene, known as the main cause of RTT, have been reported to be associated with several dysfunctions in neurodevelopment and synaptic transmission in both cultured cells and the developing mouse cortex [123].

Intellectual disability (ID) is an early-onset neurodevelopmental disorder that is defined by loss of intelligence and abnormal adaptive behavior [124, 125]. According to very recent research, X-linked intellectual disability or XLID, a type of ID caused by abnormalities in genes on the X chromosome, was found to correlate with aberrant OGT function (OGT-XLID) [126]. Importantly, *OGT* is known to be located on the X chromosome (Xq13.1) [126]. Patients with OGT-XLID showed mental and physical developmental delays. Decreased O-GlcNAcylation, decreased OGA levels, improper processing of HCF1 (host cell factor 1) by OGT, or aggregation of misfolded OGT are thought to contribute to the progression of the disease [126].

### Neurodegenerative disorders

The role of O-GlcNAcylation in AD is widely studied, and it was stated that abnormal O-GlcNAcylation is associated with either the pathogenesis or progression or both of AD [127–129]. Hyperphosphorylated Tau accumulation and amyloid-β (Aβ) plaques are the major hallmarks of AD [130–132]. Human brain Tau is shown to be O-GlcNAcylated [62], which negatively regulates tau phosphorylation [133], and interestingly, in a starved mouse model that mimics AD phenotypes, Tau phosphorylation was increased and Tau O-GlcNAcylation was decreased via low glucose uptake or metabolism [127]. In this context, several studies have used the OGA inhibitor thiamet-G to increase O-GlcNAcylation and decrease Tau phosphorylation [127]. Furthermore, a forebrain-specific *Ogt* conditional knockout mouse model showed progressive neurodegeneration with AD phenotypes. This mouse model showed excessive grooming, impaired nest building, reduced exploration, increased anxiety, and impaired amygdala-dependent cued freezing behavior in the open field and fear conditioning behavior tests, suggesting the significant role of O-GlcNAcylation in behavior symptoms in AD [134]. Similar to Tau phosphorylation, in the 5xFAD mouse model, an AD model overexpressing human mutant Aβ precursor protein, there was decreased O-GlcNAcylation and increased necroptosis in the brain, which were reversed by OGA inhibition [127, 135]. Using thiamet-G in the 5XFAD mouse model, memory and cognitive function were restored and spatial working memory and novel object recognition were improved [127, 135]. These neuroprotective effects of increased O-GlcNAcylation were also observed in other mouse models, such as JNPL3 mice [127]. Another novel OGA inhibitor, ASN90, also improved survival, breathing, and motor function in the P301L mouse model by increasing O-GlcNAcylated tau and reducing neurofibrillary tangles [136].

In PD, α-synuclein is known to be O-GlcNAcylated, and increased O-GlcNAcylation of α-synuclein by OGA inhibition has been shown to decrease α-synuclein toxicity by attenuating α-synuclein aggregation in vitro [127, 137]. In vitro studies also showed that α-synuclein aggregation is accelerated by a truncated analog of the N-terminal or NAC domain, which was attenuated when α-synuclein was O-GlcNAcylated at Thr72. O-GlcNAcylation of α-synuclein suppresses its oligomerization by disrupting hydrogen bond formation between synuclein monomers due to the extra O-linked glycosyl group [138]. Dopaminergic neuron loss and premature death were confirmed in a dopaminergic neuron-specific *Ogt* knockout, a PD mouse model, and an opposite effect was seen in *Oga* conditional knockout mouse model. The dopaminergic neuron-specific *Ogt* knockout model showed hypoactivity with defects in voluntary movements and normal motor behavior was observed in *Oga* conditional knockout mice with viral-mediated α-synuclein overexpression, suggesting that upregulated O-GlcNAcylation can fix behavioral deficits in a PD mouse model [139]. Mass spectrometry analysis identified 506 unique peptides out of 278 O-GlcNAcylated proteins and 85 peptides with 1.5-fold increases in O-GlcNAcylation levels upon thiamet G exposure, including synaptic proteins, trafficking, Notch/Wnt signaling, HDAC signaling, and circadian clock proteins. Taken together, increased O-GlcNAcylation in dopaminergic neurons may alleviate α-synuclein-mediated PD pathology through normal O-GlcNAcylation to target glycoproteins involved in the survival, maintenance, and physiological function of dopaminergic neurons. However, a contradictory study was published showing PD postmortem brains having increased O-GlcNAcylation, and in vitro study has confirmed that the inhibition of OGA by thiamet-G over a long period leads to mTOR activation, AKT phosphorylation, impaired autophagic flux, α-synuclein accumulation, and cell death. Rapamycin, mTOR inhibitor, suppressed the effects of thiamet-G, indicating that increased O-GlcNAcylation may trigger cell death instead of protecting cells. This suggests the possibility that the balance of OGT and OGA is important for managing normal dopaminergic neuronal function [140].

In HD, misfolded HTT aggregates form mutant HTT (mHTT) fibrils, which are pathogenic. In contrast to AD and PD where a decrease in O-GlcNAcylation was seen to be harming, overall reduction in O-GlcNAcylation by overexpressing OGA was shown to decrease mHTT aggregation and its toxicity by their active clearance in vitro cell culture model and fly model [117, 141]. This clearance was suggested to be a result of improved the fusion of autophagosomes with lysosomes improving the autophagic flux due to global reduction of O-GlcNAcylation [117, 141] suggesting the importance of O-GlcNacylation in regulating autophagy. Further nucleoporin (NUP), important for nucleocytoplasmic transport, was found to be misplaced in the HD mouse model (R6/2, zQ175), fly models, neurons transfected with mHTT, HD iPSC-derived neurons, and human HD brain regions [117, 142–144]. NUP is also heavily O-GlcNAcylated. Increased O-GlcNAcylation by OGA inhibition in the zQ175 mouse model restored nucleocytoplasmic transport and reduced cell death [145]. Again, an increase or decrease in O-GlcNAcylation appears to be associated with HD pathology. This indicates that there are different alterations in O-GlcNAcylation of various target glycoproteins and that O-GlcNAcylation of each target glycoprotein may lead to different consequences for HD pathology. A better understanding of the role of O-GlcNAcylation in the pathophysiology of HD will help find new avenues for therapeutic intervention.

ALS or Lou Gehrig’s disease, is a progressive neurodegenerative motor neuron disease. It is characterized by ubiquitinated aggregates, hyperphosphorylated neurofilament (NF) proteins, and the degeneration of motor neurons, brain stem nuclei, and the anterior horn of the spinal cord, resulting in focal limbic muscle weakness and progressive respiratory failure making it difficult for patients to speak, eat, breathe and move [127]. In particular, NF is increasingly being considered part of the outcome measures of ALS, and impaired O-GlcNAcylation on NF due to eOGT mutations in the ALS model is critical for NF hyperphosphorylation [146]. In SOD1 (superoxide dismutase 1)-overexpressing mouse models, decreased O-GlcNAcylation of NF and deprived O-GlcNAc immunoreactivity was found in motor neurons of the spinal cord [147]. The N*PGPx* (nonselenocysteine-containing phospholipid hydroperoxide glutathione peroxidase) knockout mouse model showed decreased O-GlcNAc levels and ROS accumulation, rescued by OGT inhibition [148]. In addition to NF, *TDP43*, one of the genes known to be involved in ubiquitinated aggregates, was also found to be O-GlcNAcylated, and increased TDP43 O-GlcNAcylation upon coexpression of OGT and hTDP-43 in the Drosophila model suppressed its aggregation and hyperphosphorylation, further leading to improved locomotion and lifespan [149]. Together, these studies indicate a crucial role for O-GlcNAcylation in regulating neurodegenerative disorders.

### Neuropsychiatric disorders

The role of O-GlcNAcylation in neuropsychiatric disorders is not widely studied, and in recent years, some researchers have shown disrupted O-GlcNAcylation in psychiatric disorders such as schizophrenia (SZ). A study showed a 253% increase in OGT mRNA levels in the superior temporal gyrus in SZ patients. Elevated O-GlcNAcylation due to increased O-GlcNAc levels has been found to lead to diabetes and mitochondrial abnormalities, which are considered SZ pathophysiology [150]. Another study showed a dampening of GABAergic currents as a result of an acute increase in O-GlcNAc, which affects the excitation/inhibition (E/I) balance. Disruption of the E/I balance found in many neuronal disorders is also a commonly seen phenomenon in SZ [151]. Therefore, the role of O-GlcNAcylation in schizophrenia and its association with behavioral symptoms will need to be further investigated.

Depression, a neuropsychiatric disorder known to be controlled by a chemical imbalance of neurotransmitters, is not just a cause of a single phenomenon. Physical and chemical causes of depression vary among individuals. As in other neuropsychiatric disorders, the role of O-GlcNAcylation in depression is not well known. In a recent study, genetically increased O-GlcNAcylation displayed antidepressant-like behaviors along with a reduction in inhibitory synaptic transmission in the mPFC [152]. In another study, conditional knockout of *Ogt* in astrocytes was used to study the role of O-GlcNAcylation in astrocytes. The results suggested that O-GlcNAcylation in astrocytes regulated many metabolic functions and ion channel activities that may regulate depressive-like disorders, suggesting the potential role of O-GlcNAcylation in depression [153]. Although it has implications that O-GlcNAcylation may have significant roles in depression, further studies are needed to fully understand the role of O-GlcNAcylation in depression, including specific cell types, brain regions, substrates, and behaviors involved. These studies will provide deeper insights into the relationship between O-GlcNAcylation and depression and may lead to the development of new treatments for this condition.

### Feeding

The role of OGT as a nutrient sensor has also been studied in relation to feeding behavior. In one study, the deletion of OGT in αCaMKII-positive neurons of the paraventricular nucleus of the hypothalamus modulates excitatory synaptic function, leading to overeating-induced obesity [154]. Another study showed that in addition to obesity, these knockout mice also showed peripheral insulin resistance and leptin receptor-expressing neuron death in the hypothalamus [155]. As a follow-up to previous research, it was shown that O-GlcNAc cycling maintained an energy balance by modulating caloric memory, suggesting the selective effect of O-GlcNAcylation on glucose homeostasis and energy balance [156].

### Other cognitive functions

Aberrant O-GlcNAcylation is also known to be responsible for several age-related diseases that affect cognitive function. In a recent study, OGT expression was found to be significantly reduced in aged mouse hippocampus which elicits aging-associated neuronal phenotypes in hippocampus such as neuronal activity and spine development. Especially, reduced OGT expression is shown to be directly associated with spatial learning and memory as well as associative fear memory. These age-related impairments in behaviors were rescued by OGT overexpression in hippocampal neurons. These data indicate that O-GlcNAcylaton is a key molecular mediator that promotes cognitive rejuvenation during aging [157]. Moreover, a study has shown that deletion of OGT in CA1 of the hippocampus impaired hippocampal-dependent fear memory consolidation due to the loss of epigenetic activity controlled by H3K27me3 [158]. Another study showed that increased O-GlcNAcylation also induced NMDA receptor and protein kinase C-independent long-term depression (LTD) at hippocampal CA3–CA1 synapses, which might be due to abnormal O-GlcNAcylation of the AMPAR GluA2 subunit. This study also confirmed that aberrant O-GlcNAcylation is associated with the impairment of hippocampus-dependent behaviors such as novel object recognition and placement memory [159]. Taken together, the results of these studies suggest that changes in O-GlcNAcylation levels can have a significant impact on learning and memory processes, and may play a role in the development of cognitive dysfunction in neurological disorders. This highlights the importance of further research in this area to better understand the underlying mechanisms and develop new treatments for neurological conditions [159].

## Conclusion

**Fig. 2:**
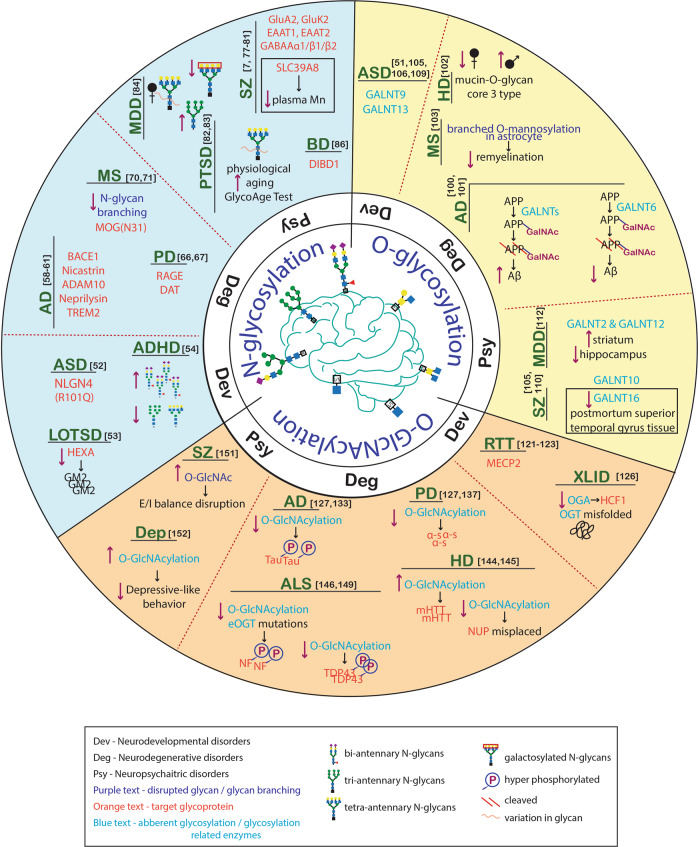
Graphic summary of glycosylation defects in several brain disorders. Among many different types of glycosylation, the most studied with regard to brain disorders are N-glycosylation, O-glycosylation, and O-GlcNAcylation. Abnormal glycosylation has been identified in various types of neurological disorders. This figure focuses on the aberrant expression of glycans or their branches (as described in this review) and aberrantly glycosylated target glycoproteins in neurodevelopmental, neurodegenerative, and neuropsychiatric disorders. Numbers enclosed in square brackets indicate the relevant references.

This review explores the role of the three primary forms of glycosylation (N-glycosylation, O-glycosylation, and O-GlcNAcylation) in the behavioral and neurological symptoms of neurodevelopmental, neurodegenerative, and neuropsychiatric disorders (Fig. 2). We found a growing body of evidence showing abnormal glycosylation in neurological disorders. However, the exact relationship between specific aspects of aberrant glycosylation and individual disorders is not yet fully understood. The factors contributing to abnormal glycosylation, such as changes in the availability of monosaccharides, glycosylation enzymes, and target glycoproteins, are complex. Therefore, comprehensive and multi-stage studies are required to fully understand the direct impact of glycosylation on disease onset and progression. Moreover, most neurological disorders result in behavioral changes, making it imperative to understand the relationship between abnormal glycosylation and behavior. To this end, interventional studies in live animals using gene knockout and knockdown systems for glycosylation-related enzymes are important for understanding the role of glycosylation in behavioral abnormalities in diseases. Additionally, it is important to combine high-throughput glycomics, proteomics, and glycoproteomics to identify aberrant glycan biosynthesis and target glycoproteins obtained from post-mortem brain samples or disease animal models. Advanced imaging techniques, such as spatial glycan MALDI imaging, are crucial in shedding light on the role of specific glycans in different brain regions that contribute to the development of neurological disorders. These studies can provide insights into the causal relationship between glycosylation and behavior, and help to establish the potential of glycosylation-based interventions as a therapeutic strategy for various neurological disorders. In conclusion, the presence of abnormal glycosylation in many CNS disorders highlights the importance of further research in this area. Studying glycosylation in depth may allow us to discover new biomarkers for neurological disorders, which could aid in early diagnosis and disease monitoring. Additionally, the understanding of the role of glycosylation in CNS disorders could lead to the development of new and effective therapies, which could greatly improve the lives of those affected by these conditions.
